# A Headache and an Abscess: A Case of Cerebral Toxoplasmosis

**DOI:** 10.7759/cureus.93678

**Published:** 2025-10-01

**Authors:** Muhammad Ghazi Khan, Alex James Francis Fischer, Makenzie Dye, Rebekah Lantz

**Affiliations:** 1 General Medicine, Wright State University, Dayton, USA

**Keywords:** brain lesions, cerebral toxoplasmosis, immunocompromised, multi-disciplinary teams, ring-enhancing lesions

## Abstract

Cerebral toxoplasmosis is a rare cause of brain abscesses, but it occurs more commonly in immunocompromised individuals. Prompt diagnosis and treatment can minimize associated neurologic mortality. When a patient presents with vague symptoms, it is important to continue gathering the patient's medical history and perform a thorough workup. This report describes a case of a 51-year-old Haitian woman who presented with seizure-like activity. Brain imaging revealed ring-enhancing lesions, leading to a new diagnosis of human immunodeficiency virus (HIV) as the cause of her immunocompromised state. Her care necessitated an interpreter, as well as a multidisciplinary team of specialists to diagnose, treat, and provide the best care.

## Introduction

Cerebral toxoplasmosis is an opportunistic brain infection caused by the obligate intracellular protozoan parasite, *Toxoplasma gondii*. This typically occurs in patients with underlying human immunodeficiency virus (HIV) infection and acquired immunodeficiency syndrome (AIDS). Traditionally, cerebral toxoplasmosis presents with constitutional symptoms such as encephalopathy, headache, focal neurological deficits, fever, or visual changes. However, in immunocompetent individuals, in about 90% of cases, the presentation is most commonly asymptomatic [1].

Toxoplasmosis, caused by *T. gondii*, is primarily transmitted through the ingestion of oocysts from contaminated soil, water, or cat feces, as well as through the consumption of raw or undercooked meat. Additional transmission routes include unpasteurized goat milk, vertical transmission (leading to congenital toxoplasmosis), organ transplantation, and blood transfusion [2-3].

After primary infection, latent tissue cysts remain dormant in organs such as the brain, skeletal muscles, and eyes. In fact, congenital cases can present with a triad of chorioretinitis, intracranial calcifications, and hydrocephalus [2]. While toxoplasmosis is prevalent in tropical climates, in the United States, it has a prevalence of 10% in adults. It is particularly significant in immunocompromised individuals, including those with HIV/AIDS, those undergoing chemotherapy, or those on immunosuppressive drugs. Also, it can reactivate during times of immunosuppression [3]. When it reactivates cerebrally, devastating neurological complications can occur, emphasizing the importance of early diagnosis and treatment.

Our patient did not have a previously established AIDS diagnosis or known exposures or hallmark symptoms of the disease. In addition, she faced a language barrier, which may have contributed to information gaps. We emphasize the importance of a high index of suspicion of cerebral toxoplasmosis in newly diagnosed AIDS individuals, as well as involving a multidisciplinary team, including internal medicine, infectious disease, neurology, neurosurgery, radiation oncology, and language interpreters when background information and diagnosis are not clear.

## Case presentation

A 51-year-old Haitian female patient, with a medical history significant for hypertension, prediabetes, thyroid-hormone-dependent hypothyroidism, and left breast cancer treated with lumpectomy, presented (in April 2024) with a headache of two-day duration, progressive altered mental status, and an unwitnessed fall. She elaborated further that she also had two weeks of subjective fever and weight loss before this. A Creole interpreter was needed for communication. It was found that she worked in an automotive factory as a machine worker, had not traveled recently but emigrated from Haiti six years ago, and was not exposed to livestock or domestic cats.

The patient’s significant other endorsed that two days ago, she had a transient period of inability to communicate with associates setting up a television in the home; this was followed by tonic-clonic movements in the four extremities and biting her tongue.

The patient was afebrile but had a soft blood pressure at 107/54 mmHg. Her heart rate was 40 beats per minute, and she was asymptomatic at this rate. Her oxygen saturation was stable on room air. Her mentation was intact, and she answered questions with clarity. She verified intermittent, generalized headaches over the past couple of days, with no alleviating or aggravating factors. Labs on presentation are shown in Table 1.

**Table 1:** Labs on presentation ALT, alanine aminotransferase; AST, aspartate aminotransferase; BUN, blood urea nitrogen; GFR, glomerular filtration rate; MCV, mean corpuscular volume; RBC, red blood count; WBC, white blood count ^*^Abnormal value

Lab test	Value	Reference range
Blood counts		
WBC	3.8	3.5-10.9 K/uL
RBC	4.25	3.95-5.26 M/uL
Hemoglobin	11.9	11.2-15.7 g/dL
Hematocrit	36.3	34.0%-49.0%
MCV	85.4	80.0-100.0 fL
Platelet count	259	140-400 K/uL
% Neutrophils	58.9	42.0%-80.0%
% Lymphocytes	36.9	14.0%-51.0%
% Monocytes	3.9*	4.0%-12.0%
% Eosinophils	0.0	0.0%-5.0%
% Basophils	0.0	0.0%-2.0%
% Immature granulocyte	0.3	<1.0%
Absolute neutrophils	2.3	1.8-7.5 K/uL
Blood chemical panel		
Sodium	138	135-148 mEq/L
Potassium	4.3	3.4-5.3 mEq/L
Carbon dioxide	23	19-32 mEq/L
BUN	18	3-29 mg/dL
Creatinine	0.5	0.5-1.2 mg/dL
Glucose	157*	70-99 mg/dL
Calcium	9.7	8.5-10.5 mg/dL
AST	19	0-46 U/L
ALT	15	0-60 U/L
Alkaline phosphatase	63	23-144 U/L
Bilirubin, total	0.2	0.0-1.2 mg/dL
Albumin	4.2	3.5-5.2 g/dL
Anion gap	13	5-15
BUN/creatinine ratio	36*	7-25
Estimated GFR	114	>60 ml/min/1.73 m^2^

She had minor solitary lesions on both legs, a <1 cm lesion on her right upper anterior thigh with scant drainage, and a 5 x 5 mm wound on her left upper medial thigh, both without tenderness, induration, or erythema. There were also shallow stage II presacral pressure ulcers.

**Figure 1:**
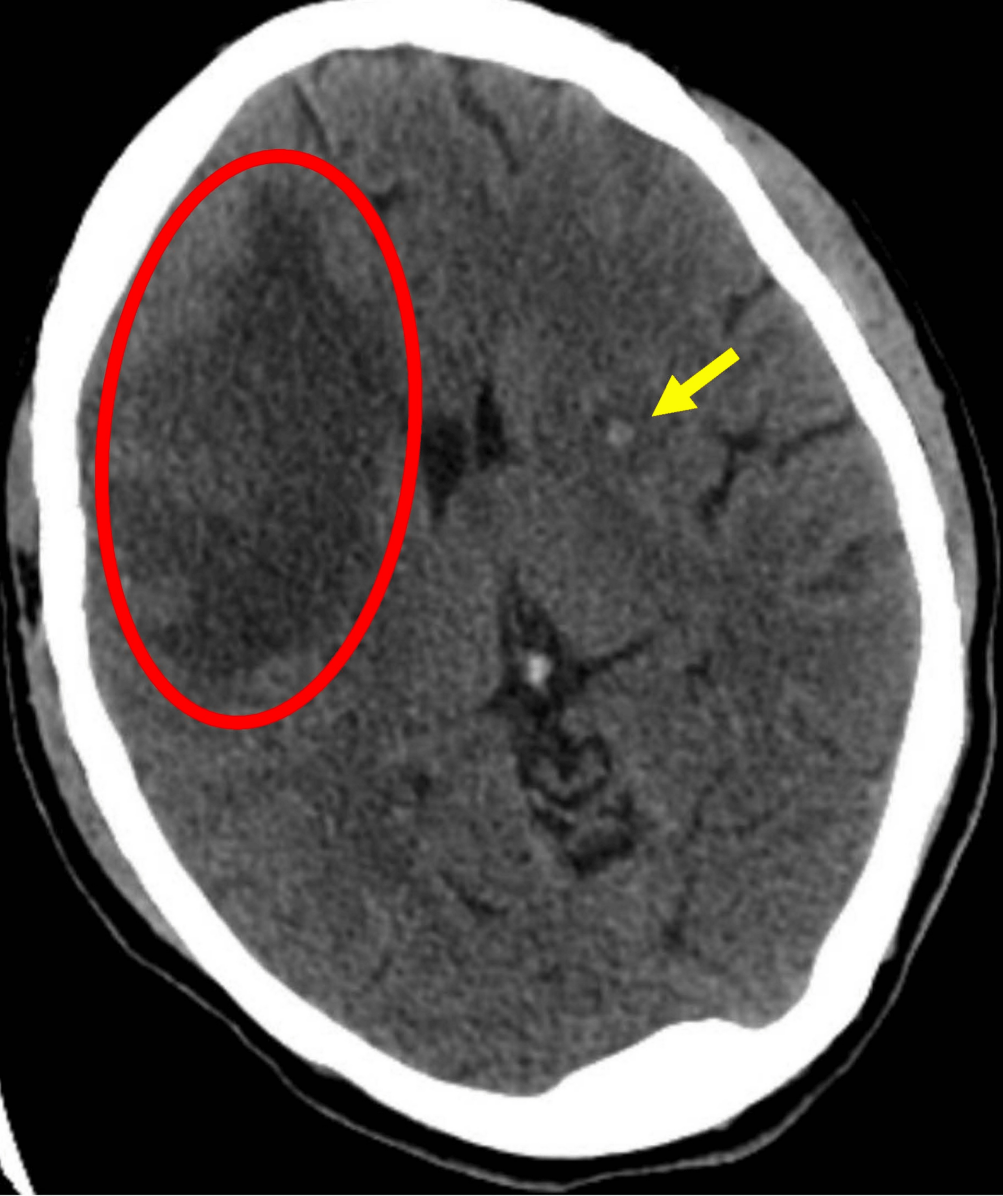
Initial head CT The yellow arrow indicates a 4 mm lesion in the left basal ganglia. The red circle represents a region of edema in the right frontal lobe.

**Figure 2:**
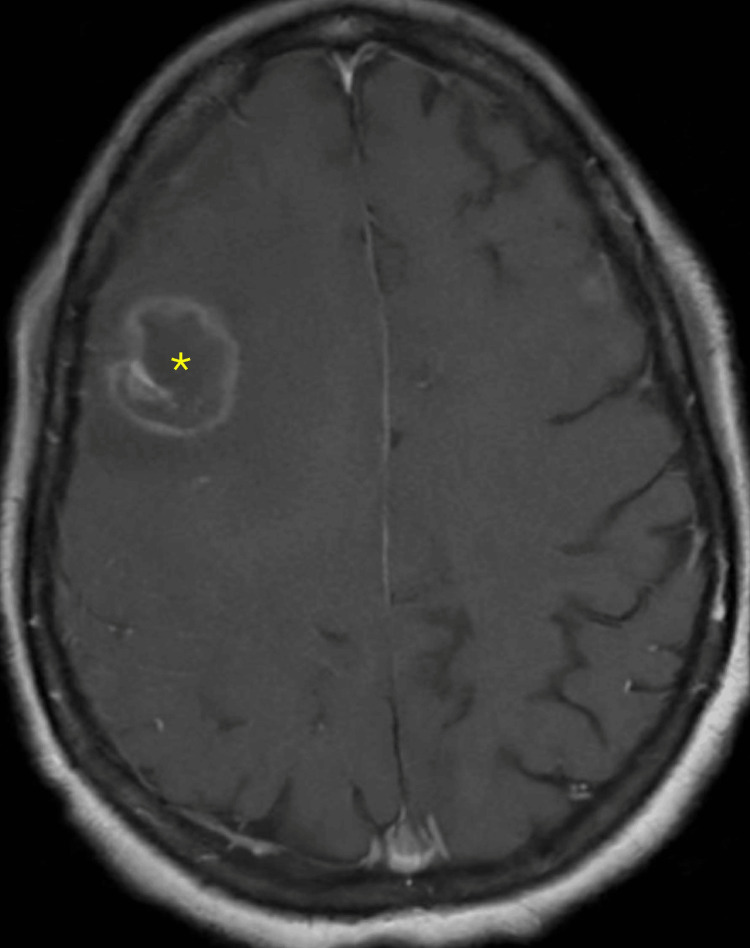
Initial brain MRI with and without contrast A ring-enhancing lesion with edema and mass effect is indicated by the yellow asterisk.

No source of infection was seen on chest X-ray, although mild cardiomegaly was present. Head CT was concerning for a 4 mm lesion in the left basal ganglia with vasogenic edema as well as a region of edema in the right frontal lobe (Figure 1). Brain MRI with and without contrast was used for further characterization and showed six intra-axial ring-enhancing lesions, along with indeterminate areas of non-enhancing post-contrast fluid-attenuated inversion recovery (FLAIR) hyperintensity at the left putamen and left lentiform nucleus/corona radiata. The largest measured 2.7 x 2.3 cm, with edema and mass effect, a 3 mm midline shift, and an eccentric target sign (Figure 2). Some lesions showed acute diffusion restriction, suggesting acute infarction. A routine electroencephalogram (EEG) showed no seizure activity. It was considered that she may need a biopsy or resection, so she was transferred to a tertiary center for further specialist evaluation.

At the tertiary hospital, Neurology was asked to assess for new-onset seizure-like activity, thought to be related to structural lesions. Routine EEG was consistent with a diagnosis of encephalopathy with focal subcortical cerebral dysfunction, likely due to an underlying structural lesion. Levetiracetam 500 mg twice daily was continued that had been initiated in the emergency room.

An infectious disease specialist was consulted to assist with the question of cerebral toxoplasmosis, given the appearance of brain lesions. Otherwise, there would be concern for primary central nervous system (CNS) lymphoma or malignant metastatic disease. Fungal cryptococcosis, blastomycosis, histoplasmosis, and tuberculosis (TB) were considered, as were bacterial nocardiosis and parasitic neurocysticercosis. Typically, these would be associated with fever and ultimately ruled out with serologies. The lab test showed HIV-1 positive status, with a viral load of 284,000 copies/mL and a CD4 T-cell count of 14/mcL (3%), consistent with a diagnosis of AIDS. Downstream tests of rapid plasma reagin (RPR), TB interferon-gamma release assay (IGRA), hepatitis panel, Cytomegalovirus (CMV) IgG, HLA B5701, G6PD, and strongyloidiasis antibodies were negative, but hepatitis A IgG tested positive. Lab results are shown in Table 2. She was recommended to initiate antibiotic therapy and continue it for two weeks. She was then to start bictegravir/emtricitabine/tenofovir alafenamide (Biktarvy) as the highly active antiretroviral therapy (HAART) agent. Fluconazole 200 mg daily was added as she had thrush.

**Table 2:** Infectious disease serologies and profile Ab, antibody; Ag, antigen; ELISA, enzyme-linked immunosorbent assay; G6PD, glucose-6-phosphodehydrogenase; Hep, hepatitis; Hgb, hemoglobin; HIV, human immunodeficiency virus; HLA, human leukocyte antigen; IgG, immunoglobulin G; IgM, immunoglobulin M; PCR, polymerase chain reaction; Quant, quantitative test; UR, urine; CMV, Cytomegalovirus; RNA, ribonucleic acid; RPR, rapid plasma reagin; TB, tuberculosis ^*^Abnormal value

Lab test	Value	Reference range
HIV Ag/Ab screen	Repeatedly reactive*	Non-reactive
HIV 1 confirmation	Positive*	Non-reactive
HIV 2 confirmation	Non-reactive	Non-reactive
% Helper cells	3*	32%-61%
HIV 1 RNA, Quant PCR	284,000*	Not detected copies/mL
HIV 1 RNA, Quant PCR	5.45*	Not detected log copies/mL
Total helper cells	14*	384-2196 mm^3^
Toxoplasma IgM Ab	<8.00	<8.00 negative
Toxoplasma IgG Ab	245*	<7.20 negative
Histoplasma UR Ag	<1	<1 negative
Histoplasma serum Ag	<1	<1 negative
Strongyloides IgG Ab ELISA	Negative	Negative
CMV IgG Ab	3.08*	Negative
Varicella zoster IgG	2174*	<135 negative
RPR (syphilis)	Non-reactive	Non-reactive
Hep A IgG Ab	Positive*	Non-reactive
Hep B surface Ag	Negative	Negative
Hep B surface Ab	Negative	Negative
HEP B core Ab	Negative	<8.0 mIU/mL negative
Hep C Ab	Negative	Negative
Mycobacterium TB	Negative	Negative
Safety profile		
G6PD	19.7	7.0-20.5 U/g Hgb
HLA B*5701 typing	Negative	Negative

Neurosurgery was requested to evaluate the patient for biopsy versus resection. She underwent a stealth-guided right frontal craniotomy for resection of the right frontal mass, with the possibility of external ventricular drain placement. A 0.9 x 0.8 x 0.4 cm frozen tissue sample of the right frontal mass was necrotic and had acute inflammation. The immunohistochemistry for this sample was positive for Toxoplasma. It was important to maintain the blood pressure below 135 mmHg systolic; levetiracetam was continued for seizure prophylaxis, and dexamethasone was added to reduce brain swelling. Biopsy reports confirmed toxoplasmosis, and the patient was started on double-strength trimethoprim/sulfamethoxazole (TMP-SMX) 160-800 mg twice daily for six-week duration. TMP/SMX was chosen due to its availability and lower cost. Again, the HIV agent was to be added two weeks after antibiotic initiation, and this was to reduce the risk of immune reconstitution inflammatory syndrome (IRIS).

At discharge, she was educated not to drive until medically cleared by Neurology; a follow-up head CT in two weeks and a brain MRI in four to six weeks were recommended.

**Figure 3:**
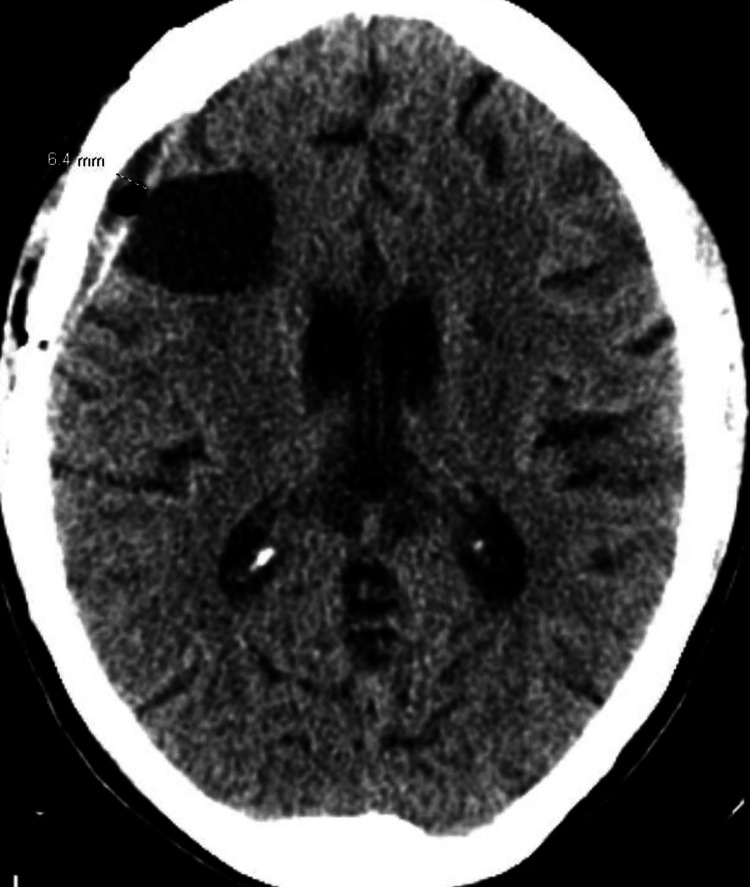
Post-craniotomy head CT without contrast Postoperative changes are evident, with an interval decrease in vasogenic edema around the resection cavity and a residual 6.4 mm fluid collection.

At her two-week follow-up appointment in May 2024, she complained of right eye pain and blurred vision with frontal headaches, worse in the morning. Her glasses had broken, and there were no focal neurologic complaints or findings on examination. Her cranial incision site was clean and with good approximation and healing. Her two-week post-craniotomy CT is shown in Figure 3.

Three weeks later, at the Infectious Diseases (ID) consultation, her thrush was found to be resolved. Different wounds and ulcerations were reported, or visualized as healed, and she had only a couple more days of TMP-SMX remaining. As per the specialist, she was cleared to start HAART. She requested to return to work.

**Figure 4:**
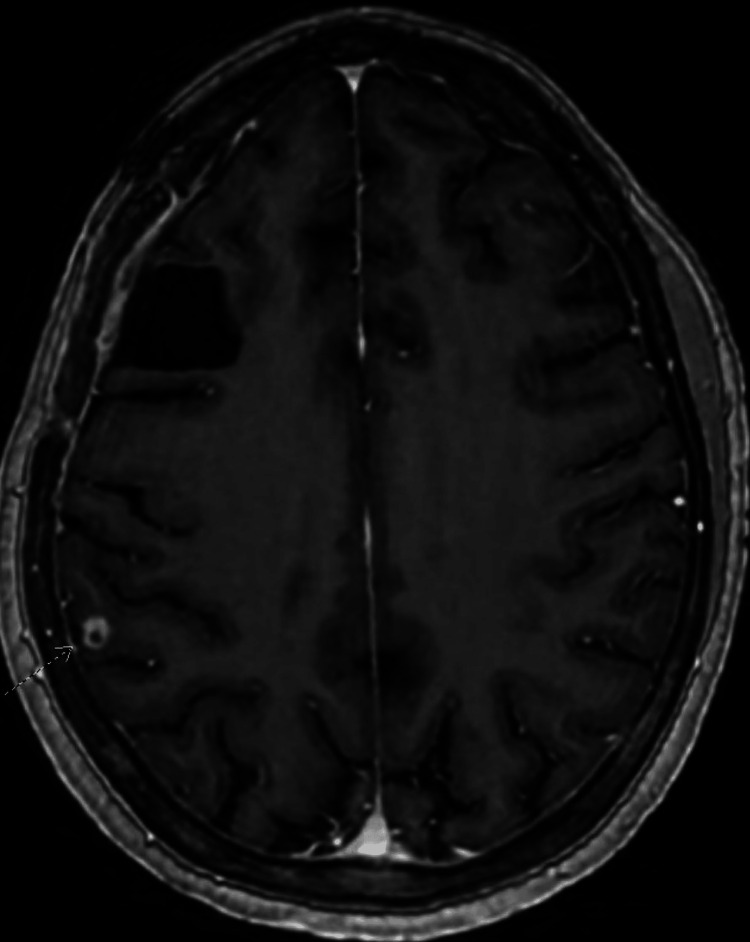
Follow-up brain MRI with and without contrast at four months after craniotomy There are resection changes in the right frontal lobe with minimal adjacent edema and numerous subcentimeter enhancing lesions with a decrease in size. These are noted by the radiologist's dotted arrow. Findings indicate a positive treatment response.

In July 2024, she had still not returned to work. The ID specialist noted an eczematous rash for which a steroid cream prescription was provided. There was no sign of mucosal involvement or concern for Stevens-Johnson syndrome (SJS) or toxic epidermal necrolysis (TEN). Figure 4 shows her brain MRI at four months.

The last follow-up with the patient was in February 2025; she continues to take Biktarvy, given her low CD4 counts of 308 mm^3^ and 15% (respective reference ranges: 384-2196 mm^3^ and 32%-61%).

## Discussion

The clinical presentation of toxoplasmosis varies based on the patient's immune status. In immunocompetent individuals, 90% of cases are asymptomatic, while the remaining 10% may exhibit mild mononucleosis-like symptoms such as fever, malaise, fatigue, acute pharyngitis, tonsillitis, lymphadenopathy, and splenomegaly [1]. The most common clinical manifestation of acute toxoplasmosis is bilateral, symmetrical, non-tender cervical adenopathy [1]. In severe cases, it can progress to cerebral toxoplasmosis, presenting with encephalopathy, focal neurological deficits, headache, altered mental status, or, in some instances, toxoplasmic chorioretinitis, which presents with eye pain and reduced vision. Rarely, immunocompetent patients may exhibit atypical presentations such as pneumonitis, acute respiratory distress syndrome (ARDS), myocarditis, pericarditis, and polymyositis [4-5]. The diagnosis of toxoplasmosis should be considered in immunocompetent individuals presenting with an acute onset of fever and lymphadenopathy. The incubation period for toxoplasmosis ranges from approximately five days to three weeks [1].

In contrast, immunosuppressed patients, particularly those who are HIV-positive and not receiving appropriate prophylaxis, are at higher risk, especially when the CD4 count falls below 100 cells/μL [4,6]. Cerebral toxoplasmosis is the most common neurological complication in individuals with AIDS, resulting from the reactivation of latent *T. gondii* cysts in immunocompromised individuals [7]. Reactivation occurs primarily when CD4 counts fall below 100 cells/μL, with the CNS being the most frequently affected site [7]. Common symptoms include encephalopathy, fever, headache, altered mental status, seizures, and focal neurological deficits. The absence of prophylactic treatment or HAART significantly increases reactivation risk, with rates as high as 30% among seropositive individuals with low CD4 counts who are not on prophylaxis. The introduction of ART has led to a dramatic decline in the incidence of toxoplasmic encephalitis. Hospitalizations in the United States for toxoplasmosis peaked in 1995 at over 10,000 cases but dropped to 3643 by 2001, emphasizing the impact of ART in reducing opportunistic infections [8]. In 2021, toxoplasmosis remains reportable in eight states: Arkansas, Delaware, Hawaii, Kentucky, Minnesota, Nebraska, Pennsylvania, and Wisconsin [9].

Besides the immune competence of an individual, the genotype of *T. gondii* can significantly influence the clinical presentation of toxoplasmosis. For example, in Europe, where genotype II is predominant, 80%-90% of infected individuals remain asymptomatic. In contrast, in South and Central America, where other genotypes are more prevalent, the infection is associated with increased disease severity [10]. There are three main *T. gondii* genotypes, types I, II, and III, each prevalent in different geographic regions, along with other atypical genotypes. In North America, genotypes I and II are the most common [10].

Diagnostic evaluation begins with serological testing. Imaging studies, such as contrast-enhanced CT or MRI, often show multiple ring-enhancing lesions in the basal ganglia and subcortical white matter, characteristic of Toxoplasma brain abscesses. Definitive diagnosis relies on PCR testing to detect Toxoplasma DNA in blood, cerebrospinal fluid (CSF), or tissue samples. When non-invasive tests are inconclusive, a histopathological examination of tissue biopsies can confirm the presence of tachyzoites or cysts, solidifying the diagnosis. These comprehensive diagnostic tools are critical for the accurate identification of cerebral toxoplasmosis [11].

Ocular toxoplasmosis is primarily diagnosed clinically, with fundoscopy playing a key role. Typical findings include yellow-white retinal lesions and the characteristic "headlight in the fog" appearance due to a marked vitreous reaction [12]. A presumptive diagnosis of toxoplasmic encephalitis can be made in patients with a CD4 count below 100 cells/μL, no prophylaxis, and the following criteria: compatible neurological symptoms (e.g., headache), positive *T. gondii* IgG antibodies, and MRI findings of multiple ring-enhancing brain lesions [13]. When these factors align, the likelihood of the diagnosis being toxoplasmic encephalitis rises to 90% [13].

Treatment for toxoplasmosis is mainly recommended for immunosuppressed individuals and pregnant women. In immunocompetent, nonpregnant adults, acute toxoplasmosis is usually self-limiting, requiring treatment only in severe cases like ocular toxoplasmosis [13]. The standard treatment for cerebral toxoplasmosis includes pyrimethamine, sulfadiazine, and leucovorin [13]. TMP/SMX is considered as effective as the traditional combination, with potentially better tolerability, though evidence for its use in immunocompetent patients is limited. For patients with sulfonamide allergies, atovaquone is an alternative [7]. For pregnant patients, spiramycin is the preferred treatment, particularly if administered before the 18th week of gestation [7].

The treatment for toxoplasmic encephalitis typically spans at least six weeks, followed by chronic maintenance therapy, which is usually initiated concurrently with or following the resolution of the acute disease flare. Adjunctive corticosteroids, such as dexamethasone, may be considered only for patients experiencing mass effects due to focal brain lesions or edema, particularly those with radiographic evidence of midline shift [7]. These steroids are generally tapered over several days. However, it is crucial to withhold glucocorticoids until primary CNS lymphoma is ruled out, as they can distort neuroimaging results and biopsy findings, potentially delaying an accurate diagnosis. In the case of toxoplasmic encephalitis, clinical improvement is typically observed within the first two weeks of therapy [7]. If there is no noticeable improvement, either clinically or radiographically, within 10 to 14 days of starting empirical treatment, the possibility of an alternative diagnosis must be considered. In such instances, a brain biopsy should be strongly considered to investigate the underlying cause further [7].

Comparatively, the treatment of ocular toxoplasmosis closely mirrors that of cerebral toxoplasmosis, with the primary distinction being the variation in dosages. Additionally, the treatment duration for ocular toxoplasmosis is generally shorter, ranging from four to six weeks [14]. Glucocorticoids may be considered after the initial course of antibiotic therapy to manage inflammation [14].

Prevention of toxoplasmosis involves several key measures. It is essential to avoid consuming raw, undercooked, or cured meats and to wash hands thoroughly after handling soil. Additionally, avoiding contact with cat litter is advised, especially in pregnant or immunocompromised individuals. For primary prophylaxis in HIV patients with a CD4 count below 100 cells/μL, the recommended regimen is TMP-SMX, one double-strength tablet (800 mg/160 mg) daily [7,11]. If TMP-SMX is not tolerated, an alternative regimen includes dapsone (50 mg daily), pyrimethamine (50 mg weekly), and leucovorin (25 mg weekly) [7,11]. Patients on antiretroviral therapy can safely discontinue primary prophylaxis for toxoplasmosis if their HIV viral load is suppressed and their CD4 count remains above 200 cells/μL for at least three months [7,11].

## Conclusions

Cerebral toxoplasmosis is a significant concern in immunocompromised patients, where the reactivation of latent *Toxoplasma gondii* cysts can lead to severe neurological complications. In this patient, timely intervention with appropriate antimicrobial therapy was key to addressing the infection. This case emphasizes the importance of considering cerebral toxoplasmosis in the differential diagnosis of a patient like ours, who was unknowingly immunocompromised. This is especially true for those presenting with neurological symptoms and where imaging findings are consistent with brain abscesses. Early recognition, a comprehensive diagnostic workup, prompt initiation of treatment, and collaborative care can significantly improve the prognosis for patients with cerebral toxoplasmosis.

## References

[1] Goldman L, Schafer AI (2024). 320. Toxoplasmosis. Goldman-Cecil Medicine.

[2] Wehbe K, Pencole L, Lhuaire M, Sibiude J, Mandelbrot L, Villena I, Picone O (2022). Hygiene measures as primary prevention of toxoplasmosis during pregnancy: a systematic review. J Gynecol Obstet Hum Reprod.

[3] Montoya JG, Liesenfeld O (2004). Toxoplasmosis. Lancet.

[4] Kim K, Weiss LM, Tanowitz HB (2016). Parasitic infections. Murray and Nadel’s Textbook of Respiratory Medicine (Sixth Edition).

[5] Pergola G, Cascone A, Russo M (2010). Acute pericarditis and myocarditis by Toxoplasma gondii in an immunocompetent young man: a case report. Infez Med.

[6] Ayoade F, Joel Chandranesan AS (2025). HIV-1-associated toxoplasmosis. StatPearls [Internet].

[7] (2025). Guidelines for the Prevention and Treatment of Opportunistic Infections in Adults and Adolescents With HIV: Toxoplasmosis. https://clinicalinfo.hiv.gov/en/guidelines/hiv-clinical-guidelines-adult-and-adolescent-opportunistic-infections/toxoplasmosis.

[8] Jones JL, Roberts JM (2012). Toxoplasmosis hospitalizations in the United States, 2008, and trends, 1993-2008. Clin Infect Dis.

[9] McCall J, Rothfeldt L, Giesbrecht K (2022). Public health surveillance and reporting for human toxoplasmosis. MMWR Morb Mortal Wkly Rep.

[10] Ferreira IM, Vidal JE, de Mattos Cde C, de Mattos LC, Qu D, Su C, Pereira-Chioccola VL (2011). Toxoplasma gondii isolates: multilocus RFLP-PCR genotyping from human patients in Sao Paulo State, Brazil identified distinct genotypes. Exp Parasitol.

[11] Robert-Gangneux F, Dardé ML (2012). Epidemiology of and diagnostic strategies for toxoplasmosis. Clin Microbiol Rev.

[12] Panda KG, Kelgaonkar A (2024). “Headlight in the fog” fundus appearance: is just a sign and not a conclusion. Oman J Ophthalmol.

[13] Dunay IR, Gajurel K, Dhakal R, Liesenfeld O, Montoya JG (2018). Treatment of toxoplasmosis: historical perspective, animal models, and current clinical practice. Clin Microbiol Rev.

[14] Garweg JG, Pleyer U (2021). Treatment strategy in human ocular toxoplasmosis: why antibiotics have failed. J Clin Med.

